# The BH3-mimetic gossypol and noncytotoxic doses of valproic acid induce apoptosis by suppressing cyclin-A2/Akt/FOXO3a signaling

**DOI:** 10.18632/oncotarget.5731

**Published:** 2015-10-16

**Authors:** Gao-Xiang Zhao, Li-Hui Xu, Hao Pan, Qiu-Ru Lin, Mei-Yun Huang, Ji-Ye Cai, Dong-Yun Ouyang, Xian-Hui He

**Affiliations:** ^1^ Department of Immunobiology, College of Life Science and Technology, Jinan University, Guangzhou, China; ^2^ Department of Cell Biology, College of Life Science and Technology, Jinan University, Guangzhou, China; ^3^ Department of Chemistry, College of Life Science and Technology, Jinan University, Guangzhou, China

**Keywords:** gossypol, valproic acid, apoptosis, akt, FOXO3a

## Abstract

Previously we reported that valproic acid (VPA) acts in synergy with GOS to enhance cell death in human DU145 cells. However, the underlying mechanism remains elusive. In this study, we observed that such synergistic cytotoxicity of GOS and VPA could be extended to human A375, HeLa, and PC-3 cancer cells. GOS and VPA co-treatment induced robust apoptosis as evidenced by caspase-8/-9/-3 activation, PARP cleavage, and nuclear fragmentation. GOS and VPA also markedly decreased cyclin A2 protein expression. Owing to the reduction of cyclin A2, Akt signaling was suppressed, leading to dephosphorylation of FOXO3a. Consequently, FOXO3a was activated and the expression of its target genes, including pro-apoptotic *FasL* and *Bim*, was upregulated. Supporting this, FOXO3a knockdown attenuated *FasL* and *Bim* upregulation and apoptosis induction in GOS+VPA-treated cells. Furthermore, blocking proteasome activity by MG132 prevented the downregulation of cyclin A2, dephosphorylation of Akt and FOXO3a, and induction of apoptosis in cells co-treated with GOS and VPA. In mouse model, GOS and VPA combination significantly inhibited the growth of A375 melanoma xenografts. Our findings indicate that GOS and VPA co-treatment induces apoptosis in human cancer cells by suppressing the cyclin-A2/Akt/FOXO3a pathway.

## INTRODUCTION

Induction of apoptosis is a common feature of successful chemotherapy and radiotherapy for various types of cancers. However, resistance to these therapies is often induced in clinical treatment of cancer, partly due to defects of pro-apoptotic signaling pathways and overexpression of anti-apoptotic genes in cancer cells [1, 2]. Many strategies have been explored to overcome such resistance of cancer. The combination of conventional chemotherapeutic agents with compounds targeting epigenetic mechanisms (i.e., epi-drugs), including inhibitors of histone deacetylases (HDACs) and DNA methyltransferases, represents a promising strategy to counteract cancer resistance [3]. Among these epi-drugs, HDAC inhibitors have garnered much interest particularly for their potential to enhance the anticancer efficacy of chemotherapy or radiotherapy [4–7].

Gossypol (GOS), a natural product isolated from cottonseeds, has been identified as a BH3-mimetic small-molecular inhibitor of Bcl-2 family members [8, 9]. Many studies have highlighted the anti-proliferative and pro-apoptotic activities of GOS in cancers, including leukemia, colon carcinoma, and prostate carcinoma cells [10–12]. However, a phase II clinical trial showed that GOS was not active in patients with recurrent chemo-sensitive small cell lung cancer and failed to induce apoptosis *in vivo* [13]. Indeed, GOS is a relatively low-toxic agent with limited cytotoxicity on cancer cells, thus limiting its use alone as an effective anticancer agent [14]. Interestingly, several studies indicated that combination therapy with GOS may induce synergistic cell death in cancer cells [15–17].

We have previously shown that GOS acts in synergy with valproic acid (VPA, a HDAC inhibitor) to induce cell death in human DU145 prostate cancer cells [18], but the precise molecular mechanism underlying such an effect is still barely understood. For example, it is unclear whether combined GOS and VPA treatment induces apoptosis in cancer cells. It is also unknown whether the synergistic effect of GOS and VPA is cancer-type specific and whether other HDAC inhibitors have similar effects when they are combined with GOS. To address these issues, we analyzed the combined effects of GOS with VPA, suberoylanilide hydroxamic acid (SAHA, also known as Vorinostat) or tubacin, and explored the potential action mechanism of GOS and VPA combination in human A375 melanoma, HeLa cervical, and PC-3 prostate cancer cells. Our data indicate that VPA, but not SAHA or tubacin, acts in synergy with GOS to induce apoptosis in these cancer cells by suppressing the cyclin-A2/Akt/FOXO3a signaling pathway.

## RESULTS

### GOS and noncytotoxic-dose VPA synergistically inhibit cancer cell proliferation

**Figure 1:**
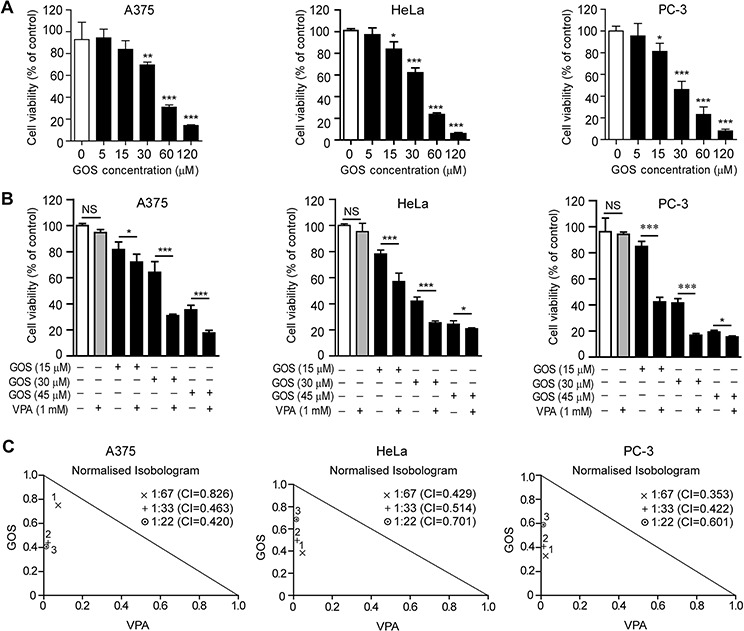
Gossypol (GOS) and valproic acid (VPA) co-treatment exhibited synergistic effects on the proliferation of A375, HeLa, and PC-3 cells **A.** Cells were treated with different concentrations of GOS for 24 h and analyzed with WST-1 assay. Data are presented as mean ± S.D. (*n* = 3). *P < 0.05; **P < 0.01; ***P < 0.001 versus control. **B.** Cells were treated with different concentrations of GOS and/or 1 mM VPA for 24 h followed by WST-1 assay. NS, non-significant; *P < 0.05; ***P < 0.001. **C.** Analysis of GOS and VPA interactions by the CalcuSyn v2.0 software. Points below the trend line indicate that the interactions of drugs are synergistic.

GOS has been reported to inhibit the proliferation of various cancer cells [10, 19]. To evaluate the inhibitory effect of GOS on the growth of human A375, HeLa, and PC-3 cancer cells, we performed a WST-1 assay in cells treated with this agent for 24 h. The results showed that GOS dose-dependently inhibited the proliferation of these cells (Figure 1A). The IC_50_ values of GOS were 43.3 ± 0.7 μM, 37.1 ± 1.4 μM, and 28.5 ± 0.9 μM for A375, HeLa, and PC-3 cells, respectively.

As our previous study has shown that VPA synergistically enhances the cytotoxicity of GOS in DU145 cells [18], we currently extended our investigation to A375, HeLa, and PC-3 cells. A low dose of VPA (1 mM) alone had no significant effects on these cancer cells (Figure 1B). However, this noncytotoxic dose of VPA strongly enhanced the inhibitory effects of GOS in all these cells (Figure 1B). To further determine whether the observed effects of VPA and GOS are additive or synergistic, these data were analyzed by CalcuSyn software. The combination indexes (CI) of VPA (1 mM) in combination with three doses of GOS (15, 30, and 45 μM) were mostly between 0.3 and 0.7 (Figure 1C), indicating a strong synergy between GOS and VPA (+++, 0.3 < CI < 0.7). Moreover, to corroborate the cytotoxicity assay, GOS and VPA co-treatment resulted in more pronounced irregular morphology and cell rounding compared with each agent alone (Supplementary Figure S1). Thus, VPA could synergistically augment the cytotoxicity of GOS in A375, HeLa, and PC-3 cells without overt cell-type preference.

### Combined GOS and VPA treatment robustly induces apoptosis

**Figure 2:**
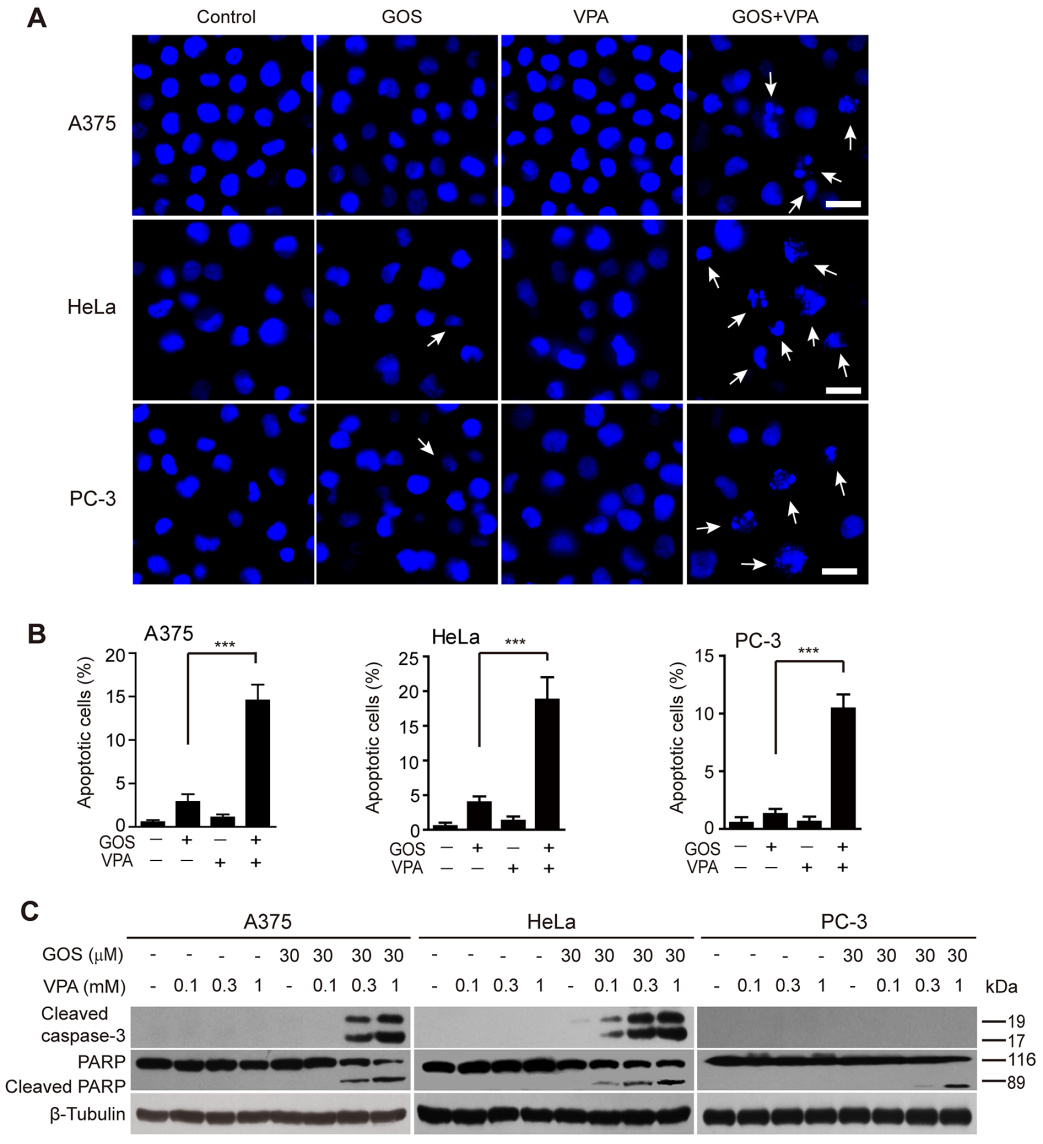
GOS and VPA synergistically induced apoptosis **A.** Cells were treated with GOS (30 μM) and/or VPA (1 mM) for 24 h, then the nuclear morphology was observed under a microscope after Hoechst 33342 staining. Arrows indicate fragmented nuclei. Scale bars, 20 μm. **B.** More than 500 cells from each sample were counted with the ZEN software (Zeiss) and the percentages of condensed or fragmented nuclei are displayed. Values are shown as mean ± S.D. (*n* = 3). ***P < 0.001. **C.** Western blot analysis of cleaved caspase-3 and PARP in cells treated with vehicle (control), VPA, GOS alone or in combination with VPA for 24 h. β-Tubulin was probed as a loading control.

Next, we sought to explore whether this synergistic cytotoxicity of GOS plus VPA resulted in apoptosis. To do this, cells were cultured overnight and then treated with GOS, VPA or their combination for 24 h. Nuclear staining showed a significant increase in condensed or fragmented nuclei in cells treated with GOS+VPA compared with each agent alone (Figure 2A and 2B). Flow cytometric analysis showed a similar increase in the rates of sub-G_0_/G_1_ DNA peaks (i.e., apoptotic peaks) in GOS+VPA-treated cells (Supplementary Figure S2A and S2B). To further explore biochemical markers of apoptosis, we assayed the activation of caspase-3. Immunoblotting revealed that GOS and VPA co-treatment robustly induced caspase-3 activation in A375 and HeLa cells in a VPA dose-dependent manner, whereas GOS or VPA alone did not (Figure 2C). However, such caspase-3 activation was barely seen in PC-3 cells, which is consistent with previous observations that caspase-7, but not caspase-3, participates in Fas-mediated apoptosis in PC-3 cells [20]. We thus analyzed the cleavage of PARP, a substrate of caspase-3/-7. As expected, GOS and VPA co-treatment induced PARP cleavage in all three cell lines (Figure 2C). Together, these data indicated that GOS and VPA synergistically induced apoptosis in all tested cells.

As GOS or VPA alone can induce macroautophagy (hereafter referred to as autophagy) in various cancer cells [21–23], we also asked whether autophagy induction had any influences on apoptosis elicited by GOS+VPA. Indeed, GOS or VPA induced autophagy as revealed by increased levels of LC3-II and co-localization of punctate LC3 with the lysosome marker LAMP2; GOS and VPA co-treatment resulted in a higher level of autophagy (Supplementary Figure S3A and S3B). To explore the influence of autophagy on GOS+VPA-induced apoptosis, we treated cells with VPA, GOS or their combination in the presence of autophagy inhibitor 3-methyladenine (3-MA) and apoptosis was quantified by flow cytometric analysis of sub-G_0_/G_1_ peak rates. Interestingly, the synergistic apoptosis was largely unaffected by 3-MA co-treatment (Supplementary Figure S3C). This suggested that autophagy might have a minimal effect on GOS + VPA-induced apoptosis in the current setting.

**Figure 3:**
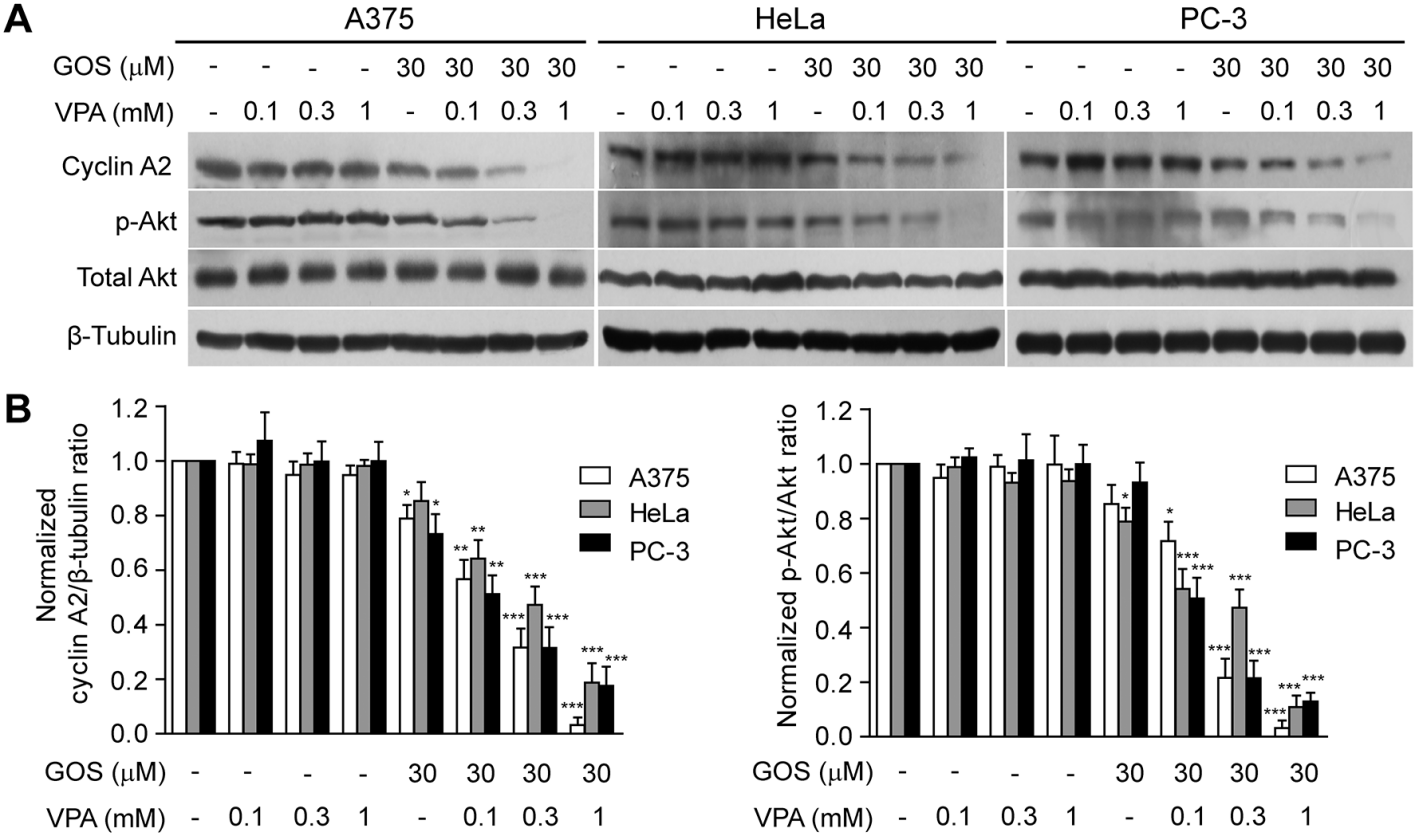
Combination of GOS and VPA induced cyclin A2 downregulation and Akt dephosphorylation **A.** Cell lysates were extracted from cells after treatment with vehicle (control), VPA, GOS, or GOS+ VPA for 24 h, and protein expression levels were analyzed by Western blotting with indicated antibodies. β-Tubulin was detected as a loading control. **B.** The densitometry ratios of cyclin A2 to β-tubulin and p-Akt to Akt normalized to control are shown in bar graph. Data are presented as mean ± S.D. (*n* = 3). *P < 0.05, **P < 0.01, and ***P < 0.001 versus control.

Although our focus was on the combined effects of GOS and VPA, we extended the combination to other HDAC inhibitors, including SAHA and tubacin. SAHA has been approved for treating cutaneous T-cell lymphoma [24] and is identified as a pan-inhibitor for class I and class II HDACs (including HDACs 1, 2, 3, and 6) [25], while tubacin is a HDAC6-selective inhibitor [26]. As expected, these inhibitors could counteract GOS-induced decrease in the acetylation levels of histones H3K9 and H4K16, with SAHA being the most effective among them (Supplementary Figure S4A). However, GOS in combination with SAHA or tubacin neither significantly increased apoptosis as compared with GOS alone (Supplementary Figure S4B) nor activated caspase-3 (Supplementary Figure S4C). These results suggested that VPA might act in synergy with GOS via a potential mechanism other than its impact on histone acetylation.

**Figure 4:**
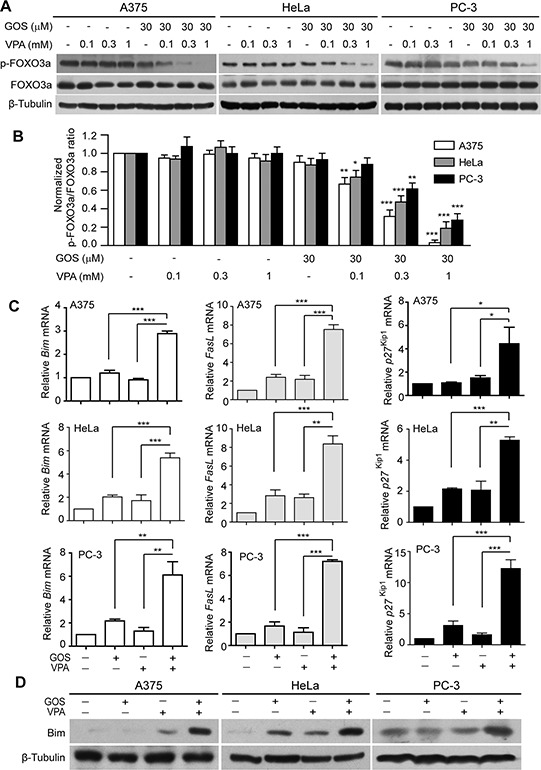
GOS and VPA co-treatment induced dephosphorylation of FOXO3a and expression of its target genes **A.** Western blot analysis of phosphorylation levels of FOXO3a in cells treated with vehicle (control), VPA, GOS, or GOS+VPA for 24 h, respectively. β-Tubulin was used as a loading control. **B.** The densitometry ratios of p-FOXO3a to FOXO3a normalized to control are shown in bar graph as mean ± S.D. (*n* = 3). *P < 0.05, **P < 0.01, and ***P < 0.001 versus control. **C.** qPCR analysis of mRNA levels of *FasL, Bim* and *p27^Kip1^* in cells treated with vehicle (control), GOS (30 μM), VPA (1 mM), or GOS+VPA. *P < 0.05; **P < 0.01; ***P < 0.001. **D.** Western blotting showing Bim expression in cells treated with vehicle (control), VPA, GOS or GOS+VPA for 24 h. β-Tubulin was used as a loading control.

### GOS and VPA co-treatment downregulates cyclin A2 levels and Akt signaling

We next sought to explore the potential action mechanism(s) for the synergistic induction of apoptosis by GOS+VPA. Given the observations that GOS affects cell cycle progression in various cancer cells [10, 27], we tested whether VPA potentiated GOS to arrest cell cycle progression. Flow cytometry showed that GOS or VPA alone arrested cells in G_0_/G_1_ and G_2_/M phase, respectively (Supplementary Figure S2C). Unexpectedly, their combination only slightly changed the ratios of cells in G_0_/G_1_ and G_2_/M phase. As cyclins play critical roles in regulating cell cycle progression, we then examined the effect of GOS+VPA on cyclin expression. Western blotting revealed that GOS+VPA co-treatment consistently and dramatically downregulated cyclin A2 expression in all tested cells in a VPA dose-dependent manner (Figure 3A and 3B).

It has been reported that cyclin A2 increases the activity of Akt by phosphorylation of specific sites at its C-terminal via cyclin-dependent kinase 2 (CDK2) activity [28] and that activated Akt can drive cellular proliferation and protect cells from apoptosis [29]. These findings prompted us to evaluate whether GOS+VPA-induced downregulation of cyclin A2 was associated with a reduction in Akt signaling. Western blotting revealed that Akt phosphorylation on Thr308 (indicative of Akt activity), but not total Akt protein, was reduced by VPA+GOS treatment in a VPA dose-dependent manner, mirroring cyclin A2 expression levels, whereas VPA or GOS alone did not have significant effects on Akt signaling or only slightly decreased it (Figure 3A and 3B). Taken together, these data indicated that GOS in combination with VPA induced the downregulation of cyclin A2, which was correlated with the decrease of Akt activity in cancer cells.

### Combined GOS and VPA treatment activates FOXO3a and its target gene expression

The forkhead FOXO family members, including FOXO3a, are important transcription factors downstream of Akt signaling [30]. In view of the fact that FOXO3a regulates the expression of pro-apoptotic genes, such as *Bim* and *FasL* [31], we next examined whether GOS in combination with VPA affected the FOXO3a signaling pathway. Western blotting showed that GOS and VPA co-treatment resulted in decreased phosphorylation of FOXO3a in a VPA dose-dependent manner in all tested cells (Figure 4A and 4B). As dephosphorylation leads to the activation of FOXO3a transcriptional activity, we subsequently detected the expression of FOXO3a target genes. Quantitative PCR (qPCR) analysis revealed that GOS and VPA co-treatment substantially increased the mRNA levels of multiple pro-apoptotic and cycle-related genes, including *Bim, FasL*, and *p27^Kip1^*, compared with each drug alone (Figure 4C). Western blot analysis showed that the protein levels of Bim were markedly increased by GOS and VPA co-treatment compared with each agent alone (Figure 4D), validating the qPCR result. As FasL and Bim can respectively activate the extrinsic and intrinsic apoptotic pathways, these data suggested that combined GOS and VPA treatment might simultaneously activate both apoptotic pathways.

### GOS and VPA co-treatment activates both intrinsic and extrinsic apoptotic pathways

**Figure 5:**
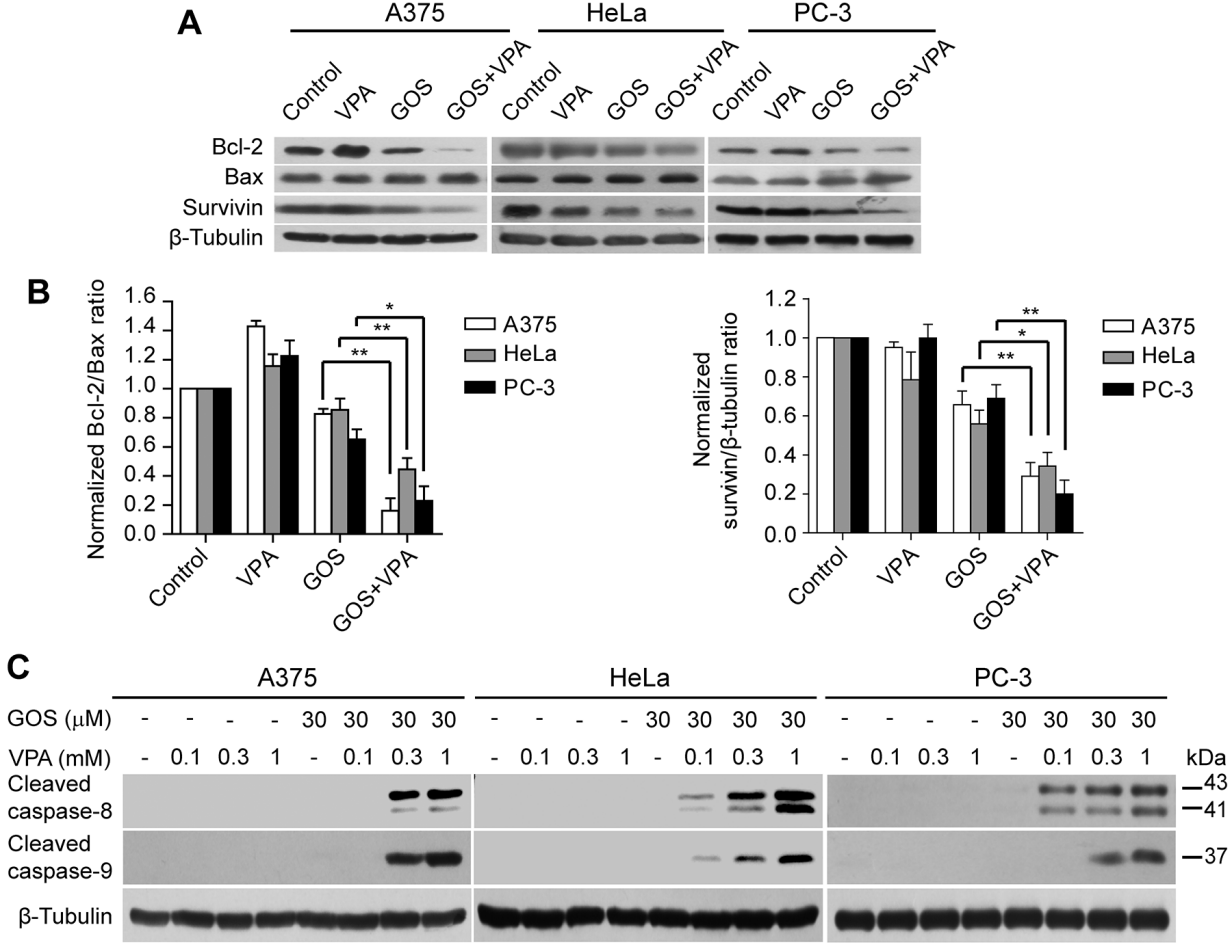
GOS and VPA co-treatment decreased the ratios of Bcl-2/Bax expression and induced caspase-8/-9 activation **A.** Western blotting was used to determine protein expression of Bcl-2, Bax and survivin in cells treated with vehicle (control), VPA (1 mM), GOS (30 μM), or GOS+VPA for 24 h. β-Tubulin was used as a loading control. **B.** The densitometry ratios of Bcl-2 to Bax or survivin to β-tubulin normalized to control are shown in bar graph as mean ± S.D. (*n* = 3). *P < 0.05; **P < 0.01. **C.** Cells were treated with vehicle (control), VPA, GOS, or GOS+VPA for 24 h, and cleaved caspase-8/-9 were determined by Western blotting. β-Tubulin was used as a loading control.

To further investigate the mechanism underlying GOS+VPA-induced apoptosis, we examined protein levels of Bcl-2 family members Bcl-2 and Bax by Western blot analysis. Co-treatment with GOS and VPA led to a significant decrease in Bcl-2 and a slight increase in Bax expression (Figure 5A). Notably, GOS and VPA co-treatment caused a marked reduction in the Bcl-2/Bax ratio (Figure 5B), which is a critical determinant of apoptosis. In addition, survivin, a member of the inhibitor of apoptosis protein (IAP) family, was also dramatically decreased by GOS+VPA co-treatment in these cells (Figure 5A and 5B). Together with increased expression of pro-apoptotic Bim, these results suggested that GOS and VPA co-treatment could activate the intrinsic apoptotic pathway.

To further validate that GOS+VPA could activate not only the intrinsic apoptotic pathway but also the extrinsic apoptotic pathway, we determined whether GOS plus VPA activated caspase-8 and caspase-9. Indeed, GOS+VPA profoundly induced caspase-8 and caspase-9 cleavage in a VPA dose-dependent manner, reflecting the activation of caspase-8 (a marker of the extrinsic pathway) and caspase-9 (a marker of the intrinsic pathway), whereas GOS or VPA alone did not (Figure 5C). Altogether, these results indicated that GOS acted in synergy with VPA to trigger both extrinsic and intrinsic apoptotic pathways in all tested cancer cells.

### FOXO3a knockdown attenuates GOS+VPA-induced activation of both apoptotic pathways

**Figure 6:**
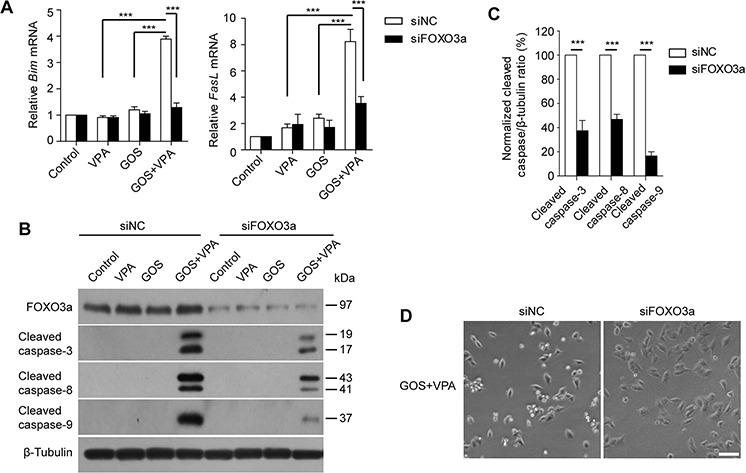
FOXO3a knockdown attenuated GOS+VPA-induced Bim and FasL expression and caspase activation A375 cells were transfected with siNC or siFOXO3a for 72 h, followed by treatment with vehicle (control), VPA (1 mM), GOS (30 μM), or GOS+VPA for 24 h, respectively. **A.** The mRNA levels of *FasL* and *Bim* was analyzed by qPCR. Data are shown as mean ± S.D. (*n* = 3). ***P < 0.001. **B.** Cell lysates were subjected to Western blot analysis for FOXO3a expression and caspase-3/-8/-9 activation. β-Tubulin was detected as a loading control. **C.** The densitometry ratios of cleaved caspase-3/-8/-9 to β-tubulin normalized to GOS+VPA-treated siNC group were shown in the bar graph. Data are presented as mean ± S.D. (*n* = 3). ***P < 0.001. **D.** Cells were treated with GOS+VPA for 24 h and their morphology was observed using phase-contrast microscopy (10 ×). Scale bar, 100 μm.

We next sought to confirm the role of FOXO3a in GOS+VPA-induced apoptosis by using small interfering RNA (siRNA). Knockdown with FOXO3a-specific siRNA resulted in more than 70% decrease in its protein levels. qPCR analysis showed that FOXO3a knockdown markedly diminished GOS+VPA-induced expression of *Bim* and *FasL* mRNAs as compared with negative control (Figure 6A). Furthermore, GOS+VPA-induced activation of caspase-8, caspase-9, and caspase-3 was dramatically attenuated by FOXO3a knockdown (Figure 6B and 6C). Knockdown of FOXO3a also decreased the extent of GOS+VPA-induced irregular morphology and cell rounding as compared with negative control (Figure 6D). These data indicated that FOXO3a had a critical role in GOS+VPA-induced activation of both extrinsic and intrinsic apoptotic pathways.

### GOS and VPA co-treatment induces cyclin A2 degradation via the ubiquitin-proteasome pathway

**Figure 7:**
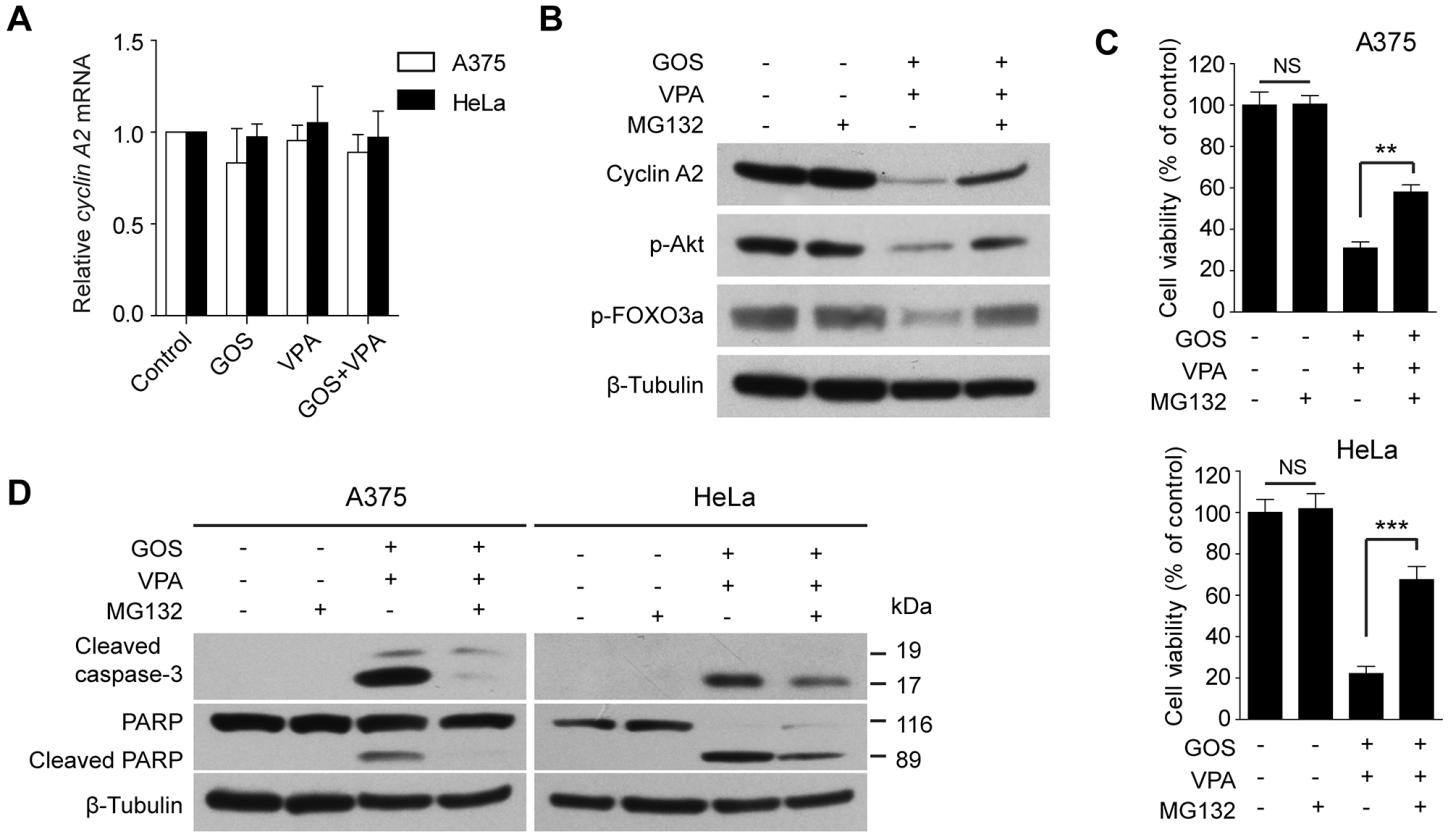
GOS and VPA co-treatment induced proteasome-dependent degradation of cyclin A2 **A.** qPCR analysis of the mRNA levels of *cyclin A2* in A375 and HeLa cells treated with vehicle (control), GOS (30 μM), VPA (1 mM), or GOS+VPA. **B.** Western blot analysis of cyclin A2, p-Akt and p-FOXO3a in A375 cells were treated with vehicle (control), MG132 (0.5 μM), GOS+VPA in the presence or absence of MG132 for 24 h. β-Tubulin was used as a loading control. **C.** A375 and HeLa cells were treated with MG132 and/or GOS+VPA for 24 h followed by WST-1 assay. Data are expressed as mean ± S.D. (*n* = 3). NS, non-significant; **P < 0.01; ***P < 0.001. **D.** Western blot analysis of cleaved caspase-3 and PARP in A375 and HeLa cells treated with vehicle (control), MG132, and GOS+VPA in the presence or absence of MG132 for 24 h. β-Tubulin was probed as a loading control.

We next explored the underlying mechanism for GOS+VPA-induced cyclin A2 downregulation. To this end, we first detected *cyclin A2* mRNA levels using qPCR assay. The mRNA levels of *cyclin A2* in GOS+VPA-treated cells were comparable to those in control, GOS- or VPA-treated cells (Figure 7A), suggesting that GOS+VPA-induced downregulation of cyclin A2 might be at the posttranscriptional level. As cyclin A2 protein levels can be regulated by post-translational modification including ubiquitination [32], we assayed whether cyclin A2 was degraded in GOS+VPA-treated cells through the ubiquitin-proteasome pathway. The proteasome inhibitor MG132 could suppress GOS+VPA-induced downregulation of cyclin A2 (Figure 7B), indicating that the ubiquitin-proteasome pathway was involved in cyclin A2 degradation. Concomitant with the restoration of cyclin A2 levels, the phosphorylation levels of Akt and FOXO3a were also reversed by MG132 (Figure 7B), corroborating the role of cyclin A2 in regulating the Akt/FOXO3a signaling axis.

Moreover, GOS+VPA-induced decrease in cell viability was also partly restored by MG132 (Figure 7C). Next, we detected caspase-3 activation and PARP cleavage by Western blotting. When A375 and HeLa cells were co-treated with GOS and VPA in the presence of MG132, the levels of cleaved caspase-3 and PARP were markedly decreased (Figure 7D). Although it has been shown to induce apoptosis in various cancer cells [33, 34], MG132 alone did not induce apoptosis in our experimental setting (Figure 7D). These data suggested that the GOS+VPA-induced cyclin A2 degradation was at least partly mediated by the ubiquitin-proteasome pathway and that this process was critical for the synergistic induction of apoptosis.

### GOS acts in synergy with VPA to inhibit tumor growth *in vivo*

**Figure 8:**
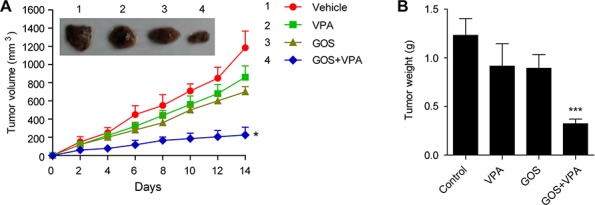
Combination of GOS and VPA significantly suppressed xenografted tumor growth *in vivo* Nude mice with A375 melanoma xenografts were administered intragastrically with vehicle (2% Tween-80 in PBS), VPA (200 mg/kg/d), GOS (20 mg/kg/d), or VPA and GOS combination for 14 consecutive days (*n* = 6 mice per group). **A.** Tumor volumes were measured once every 2 days. After 14 days, tumors were excised and representative xenografted tumors were photographed. **B.** Tumors were weighted at the end of experiments. Data are presented as mean ± S.D. (*n* = 6). *P < 0.05 and ***P < 0.001 versus control.

Finally, we used nude mice with human A375 melanoma xenografts as a model to verify the *in vivo* anticancer potency of GOS and VPA co-treatment. Administration with GOS (20 mg/kg/d) or VPA (200 mg/kg/d) alone slightly (but not significantly) decreased the growth of xenografted tumors, whereas GOS and VPA combination significantly suppressed the growth of xenografted tumors (Figure 8A and 8B). No significant weight loss and death were recorded during the course of treatment (Supplementary Figure S5). These results indicated that GOS could act in synergy with VPA to suppress the growth of human xenografted tumors in nude mice.

## DISCUSSION

**Figure 9:**
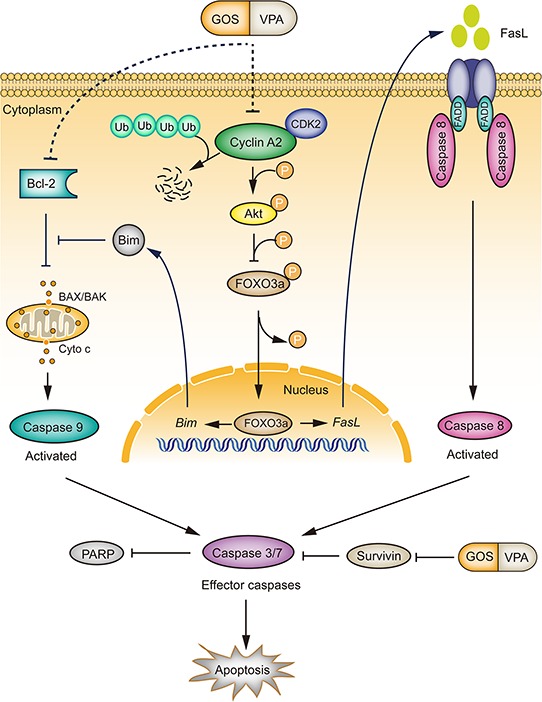
A schematic depicts the action mechanism by which GOS and VPA synergistically activate both extrinsic and intrinsic apoptotic pathways GOS and VPA co-treatment promotes cyclin A2 degradation via the ubiquitin-proteasome pathway, leading to attenuated Akt signaling. Decreased Akt signaling in turn results in dephosphorylation of FOXO3a and thereby activation of FOXO3a transcriptional activity leading to upregulation of FOXO3a target genes for apoptosis. Furthermore, GOS and VPA decrease the ratio of Bcl-2/Bax, resulting in release of cytochrome c (Cyto c) and activation of caspase-9. GOS and VPA combination also inhibits the expression of survivin. The overall outcome of GOS and VPA co-treatment is the activation of both intrinsic and extrinsic apoptotic pathways, leading to robust apoptosis. The as-yet-uncharacterized processes for GOS+VPA-induced downregulation of cyclin A2 and Bcl-2 are indicated by dotted lines.

In line with our previous observations in DU145 cells [18], we found in this study that noncytotoxic doses of VPA enhanced the cytotoxicity of GOS in human A375, HeLa, and PC-3 cancer cells, suggesting that such synergistic effects have no apparent cell-type preference. We further demonstrated that combined GOS and VPA treatment induced robust apoptosis in these cancer cells. In our experimental settings, VPA seemed to be a distinct HDAC inhibitor that could enhance the capacity of GOS to induce apoptosis. This combined action of GOS and VPA was largely mediated by suppressing the cyclin-A2/Akt/FOXO3a signaling pathway (Figure 9).

Cancer cells usually possess or acquire resistance to apoptosis, thus refractory to single-agent chemotherapy or radiotherapy [35, 36]. Consistent with this notion, we found that GOS alone could only induce low levels of apoptosis, although it significantly inhibited cell proliferation. Such resistance to apoptosis may be due to high expression levels of anti-apoptotic proteins including Bcl-2 and survivin in these cells, which remained high after GOS treatment (Figure 5A). Moreover, the high levels of Akt signaling, which promotes the survival of cancer cells [37, 38], was minimally influenced by GOS alone. However, GOS and VPA co-treatment had overcome this anti-apoptotic mechanism at least partly by decreasing the phosphorylation levels of Akt and its downstream substrate FOXO3a. It is believed that reduced phosphorylation of FOXO transcription factors leads to their activation, thus upregulating the expression of their target genes [30] but blocking the expression of survivin [39]. In line with this concept, we found that the expression of FOXO3a target genes, including pro-apoptotic *Bim* and *FasL*, were markedly increased, whereas the anti-apoptotic survivin levels were sharply decreased. It is known that FasL is a death ligand that can trigger the extrinsic apoptotic pathway through binding to its receptor Fas expressed on most cancer cells [40]. Thus, by downregulating anti-apoptotic components and upregulating pro-apoptotic proteins, GOS and VPA co-treatment induced robust activation of caspase-9, indicative of the intrinsic apoptotic pathway, and caspase-8, reflective of the extrinsic apoptotic pathway [41]. Further supporting the critical role of FOXO3a in this process, knockdown of FOXO3a significantly diminished GOS+VPA-induced expression of *Bim* and *FasL*, and attenuated the activation of both extrinsic and intrinsic apoptotic pathways. Thus, our data indicated that combined GOS and VPA treatment simultaneously triggered both apoptotic pathways by suppressing the Akt/FOXO3a signaling axis.

In keeping with previous findings that CDK2/cyclin A2 complex is a physiologic kinase governing Akt activation [28], we found that GOS+VPA-induced suppression of Akt signaling was highly correlated with the reduction of cyclin A2 levels. It is believed that cyclin A2 is degraded through the ubiquitin-proteasome pathway [32]. In line with this notion, we showed that the proteasome inhibitor MG132 significantly reversed the downregulation of cyclin A2. When such downregulation of cyclin A2 levels was reversed by MG132, the phosphorylation of Akt and FOXO3a was restored and the apoptosis was attenuated, further confirming the link between cyclin A2 and Akt. Based on these observations, it is concluded that GOS and VPA co-treatment induces apoptosis by suppressing the cyclin-A2/Akt/FOXO3a signaling pathway. In addition, these observations also suggest that cyclin A2 expression may confer cancer cells resistance to apoptosis. In support of this hypothesis, loss of cyclin A2 and CDK2 in oncogene-transformed mouse embryonic fibroblasts (MEFs) leads to cell apoptosis and suppresses their ability to form tumors in immunocompromised mice [42]. Collectively, our data indicate that GOS in combination with VPA overcomes the resistance of cancer cells to apoptosis at least partly by inducing cyclin A2 degradation and downregulating Akt signaling. Yet further research is necessary to reveal the precise mechanism underlying GOS+VPA-induced cyclin A2 degradation.

GOS has been investigated in clinic as a contraceptive for males in China several decades ago [43]. Over the past decades, GOS re-attracts scientific attention due to the finding of its anticancer activities and its tolerance to humans [44–47]. However, it is only partly effective in inducing cell death at high doses [14]. Our finding that noncytotoxic doses of VPA could strongly enhance apoptosis in cancer cells treated with GOS has clinical implications, as such effects of VPA were observed in the concentrations between 0.1 mM and 1 mM, which are achievable and tolerable *in vivo* in humans with acceptable side effects [48]. In addition, we also provided evidence showing *in vivo* inhibitory effect of combined GOS and VPA treatment on the growth of xenografted tumors. Further clinical research is thus warranted to validate our *in vitro* and animal studies.

In summary, we provided evidence that, in addition to modulating Bcl-2 family members, GOS in combination with VPA increased the expression of *FasL* and *Bim* by downregulating the cyclin-A2/Akt/FOXO3a pathway. By suppressing this pro-survival signaling pathway, GOS and VPA co-treatment may lower the apoptotic threshold, thereby rendering the chemo-resistant cancer cells more susceptible to apoptosis. Our study suggests that the combination of GOS and VPA constitutes a promising therapeutic regimen for certain human cancers with chemo-resistance.

## MATERIALS AND METHODS

### Reagents

Gossypol (GOS), valproic acid sodium salt (VPA), Tween 80, suberoylanilide hydroxamic acid (SAHA), tubacin, 3-methyladenine (3-MA), propidium iodide (PI), dimethyl sulfoxide (DMSO), Hoechst 33342, and MG132 were purchased from Sigma-Aldrich (St. Louis, MO, USA). GOS was dissolved in dimethyl sulfoxide (DMSO). The final concentration of DMSO never exceeded 0.2%, which had no cytotoxicity in this study (data not shown). WST-1 assay kit was obtained from Roche (Penzberg, Germany). RNase A, Dulbecco's modified Eagle's medium (DMEM), RPMI-1640, fetal bovine serum (FBS), penicillin and streptomycin were purchased from Invitrogen (Carlsbad, CA, USA). Polyvinylidene difluoride (PVDF) membranes (Hybond-P) were purchased from GE Healthcare Life Sciences (Piscataway, NJ, USA). The antibodies against cyclin A2 (#4656), Bcl-2 (#2870), Bax (#2772), Bim (#2933), PARP(#9532), cleaved caspase-3 (#9664), cleaved caspase-8 (#9496), cleaved caspase-9 (#7237), phospho(p)-Akt (Thr308: #2965), Akt (#2920), FOXO3a (#2497), p-FOXO3a (Ser253: #9466), acetyl-histone H3 (Lys9: #9649), acetyl-histone H4 (Lys16: #8804), histone H3 (#9717), LC3B (#3868), and β-tubulin (#2128) were obtained from Cell Signaling Technology (Danvers, MA, USA). The antibody against survivin (sc-10811) was from Santa Cruz Biotechnology (Santa Cruz, CA, USA).

### Cell culture and treatment

Human cancer A375, PC-3 and HeLa cell lines were obtained from the Cell Bank of the Chinese Academy of Sciences (Shanghai, China). A375 and HeLa Cells were maintained in DMEM while PC-3 cells were maintained in RPMI-1640, supplemented with 10% FBS, 100 U/ml penicillin and 100 μg/ml streptomycin at 37°C in a humidified incubator with 5% CO_2_ and sub-cultured every 2–3 d. In all experiments, cells in log-phase were seeded for 24 h and the medium was replaced with fresh medium with or without drugs.

### Cell viability assay

Cell viability was measured using the WST-1 assay kit according to the manufacturer's instructions. The 50% inhibition concentration (IC_50_) indicates the concentration corresponding to 50% reduction of cell proliferation as compared with control cells. Cells in log-phase were seeded in 96-well plates (3500 cells/well) for 24 h and then treated with indicated concentrations of GOS and/or VPA. After 24 h, 10 μl of WST-1 reagent was added to each well and the plates were incubated for 0.5–2 h at 37°C. The absorbance at 450 nm with a reference at 630 nm was measured by a microplate reader (Model 680; Bio-Rad, Hercules, CA).

### Cell cycle analysis

Analysis of cell cycle was performed as described previously [18]. In brief, cells were fixed with 70% ethanol and stained with phosphate-buffered saline (PBS) containing 50 μg/ml PI and 30 μg/ml RNase A. DNA content data were acquired using CELLQuest software on a flow cytometer (FACSCalibur; Becton-Dickinson, Mountain View, CA, USA). A minimum of 20,000 events was collected for each sample.

### Immunofluorescence microscopy

Immunofluorescent staining was performed as described previously [49]. Cells were fixed in 4% paraformaldehyde, permeabilized with ice-cold 100% methanol, and immunostained with LC3B and LAMP2 (ab25631; Abcam, Cambridge, MA, USA) antibodies followed by incubation with CF488A-conjugated goat-anti-mouse IgG (#20018) and CF568-conjugated goat-anti-rabbit IgG (#20103), highly cross-absorbed (Biotium, Hayward, CA, USA). Nuclei were revealed by Hoechst 33342 staining. Fluorescence images were collected under a Leica DMIRB fluorescent microscope (Leica Microsystems, Wetzlar, Germany) equipped with a Spinning Disk Confocal Microscopy system (UltraView cooled CCD; Perkin Elmer, Waltham, MA, USA).

### Quantitative PCR

Real-time quantitative PCR (qPCR) was performed on a Chromo4 four-color real-time PCR detection system (Bio-Rad). qPCR for each sample was performed twice in triplicates using a 20-μl reaction system containing 50 ng initial total RNA. Both annealing and extending temperature were set to 60°C. Forty PCR cycles were run and the melting curves were recorded. The primers used are as follows: 5′-GAAGAGAGGGAACCACAGCA-3′ (sense) and, 5′-TTGCCTGTTAAATGGGCCAC-3′ (anti-sense) for *FasL*; 5′-TCATCGCGGTATTCGGTTCG-3′ (sense) and 5′-CTTCACCTCCGTGATTGCCT-3′ (anti-sense) for *Bim*; 5′-ACGGGGTTAGCGGAGCAA-3′ (sense) and 5′-ATGTCCATTCCATGAAGTCAGC-3′ (anti-sense) for *p27^Kip1^*; 5′-CCTCCTTGGAAAGCAAA CAGTAAA-3′ (sense) and 5′-ACACTCACTGGCT TTTCATCTTC-3′ (anti-sense) for *cyclin A2*; 5′-AGAT CTGGCACCACACCTTCT-3′ (sense) and 5′-CTTTG ATGTCACGCACGATTT-3′ (anti-sense) for *β-actin*. All other parameters for the reaction system and the PCR program were set according to the manufacturer's protocol for the SYBR PrimeScript RT-PCR kit (Takara, Dalian, China). The specificity of PCR was determined by a following melting curve analysis. The relative quantification of gene expression was analyzed by the 2^−ΔΔCt^ method.

### Protein extraction

Cells were washed thoroughly with ice-cold PBS and lysed with 2 × sodium dodecyl sulfate-polyacrylamide gel electrophoresis (SDS-PAGE) loading buffer. Lysates were sonicated, boiled and clarified by centrifugation at 12,000 × g for 20 min at 4°C. Protein concentration was measured by SDS-PAGE with a known sample determined by a BCA protein assay kit (#23227; Pierce) according to the manufacturer's instructions. Samples were quickly frozen in liquid nitrogen and then stored at −80°C until use.

### Western blot analysis

Samples containing 30 μg of total protein were separated by SDS-PAGE and transferred onto a PVDF membrane. After incubation in blocking buffer (50 mM Tris-buffered saline (pH7.4) containing 5% nonfat dry milk and 0.1% Tween-20), the membranes were probed with indicated primary antibodies, followed by incubation with horseradish peroxidase (HRP)-conjugated goat anti-rabbit IgG (Jackson ImmunoResearch, #111–035-003) or goat anti-mouse IgG (Jackson ImmunoResearch, 115–035-003). Bands were revealed with an enhanced chemiluminescence kit (#P0018; Beyotime, Haimen, China) and recorded on X-ray films (Kodak, Xiamen, China). Images were captured using FluorChem 8000 imaging system (AlphaInnotech; San Leandro, CA, USA) and the densitometry of each band was quantified using AlphaEaseFC software ((AlphaInnotech).

### Small interfering RNA (siRNA)

The siRNA (5′-CCAUGUCACACUAUGGUAA-3′) duplexes targeting FOXO3a and negative control siRNA were designed and synthesized by RiboBio (Guangzhou, China). Transfection was performed using Lipofectamine RNAiMAX (Invitrogen) according to the manufacturer's instructions. In brief, one day before transfection, Cells were plated in 6-well plates at 30–50% confluency, and transfected with 50 nM siFOXO3a or negative control siRNA (siNC) for 72 h, followed by treatment with GOS, VPA or their combination for 24 h. Western blotting was used to determine protein expression levels while gene expression was quantified by qPCR.

### Treatment of xenografted tumors

Specific-pathogen-free female BALB/c (Nu/Nu) nude mice (6 weeks of age) were purchased from Guangzhou University of Traditional Chinese Medicine (Guangzhou, China) and housed under pathogen-free conditions in microisolator cages. The animals were acclimated for 1 week before experiments. Animal experiments were conducted following the National Institutes of Health guidelines and were approved by the Committee on the Ethics of Animal Experiments of Jinan University. Human melanoma A375 cells (1 × 10^6^ cells/100 μl in PBS) in the logarithmic growth phase were subcutaneously inoculated in the right flank of nude mice. When tumors were detectable (about 6 days after inoculation) (set as day 0), mice were administrated consecutively with vehicle (2% Tween-80 in PBS), VPA (200 mg/kg/d), GOS (20 mg/kg/d), or VPA and GOS combination via gavage, respectively. The dosages GOS and VPA used in this *in vivo* experiment were based on previous reports [50, 51] and our preliminary experiments, showing that combined GOS and VPA of such doses had no apparent toxic to mice. Tumor volume (V) was measured with a caliper once every 2 days and calculated with the formula V = (L × W^2^)/2, where L represents larger diameter and W represents smaller diameter. At the end of experiments, mice were euthanized by CO_2_ inhalation and tumor tissues were harvested for weighting.

### Statistical analysis

All experiments were performed at least three times, with one representative experiment shown. Data were presented as the mean ± standard deviation (S.D.). Statistical analysis was performed using GraphPad Prism 5 (GraphPad Software Inc., San Diego, CA, USA). One-way ANOVA, followed by the Tukey post-hoc test was used to analyze the statistical significance among multiple groups and a *P*-value of < 0.05 was considered statistically significant.

## SUPPLEMENTARY FIGURES


