# Supplementary material for: The BH3-mimetic gossypol and noncytotoxic doses of valproic acid induce apoptosis by suppressing cyclin-A2/Akt/FOXO3a signaling
Source: Oncotarget. 2015 Oct 16;6(36):38952–66. doi: 10.18632/oncotarget.5731 (PMC4770749; doi:10.18632/oncotarget.5731)
Supplement: Supplementary file 1 [file oncotarget-06-38952-s001.pdf]

SUPPLEMENTARY FIGURES

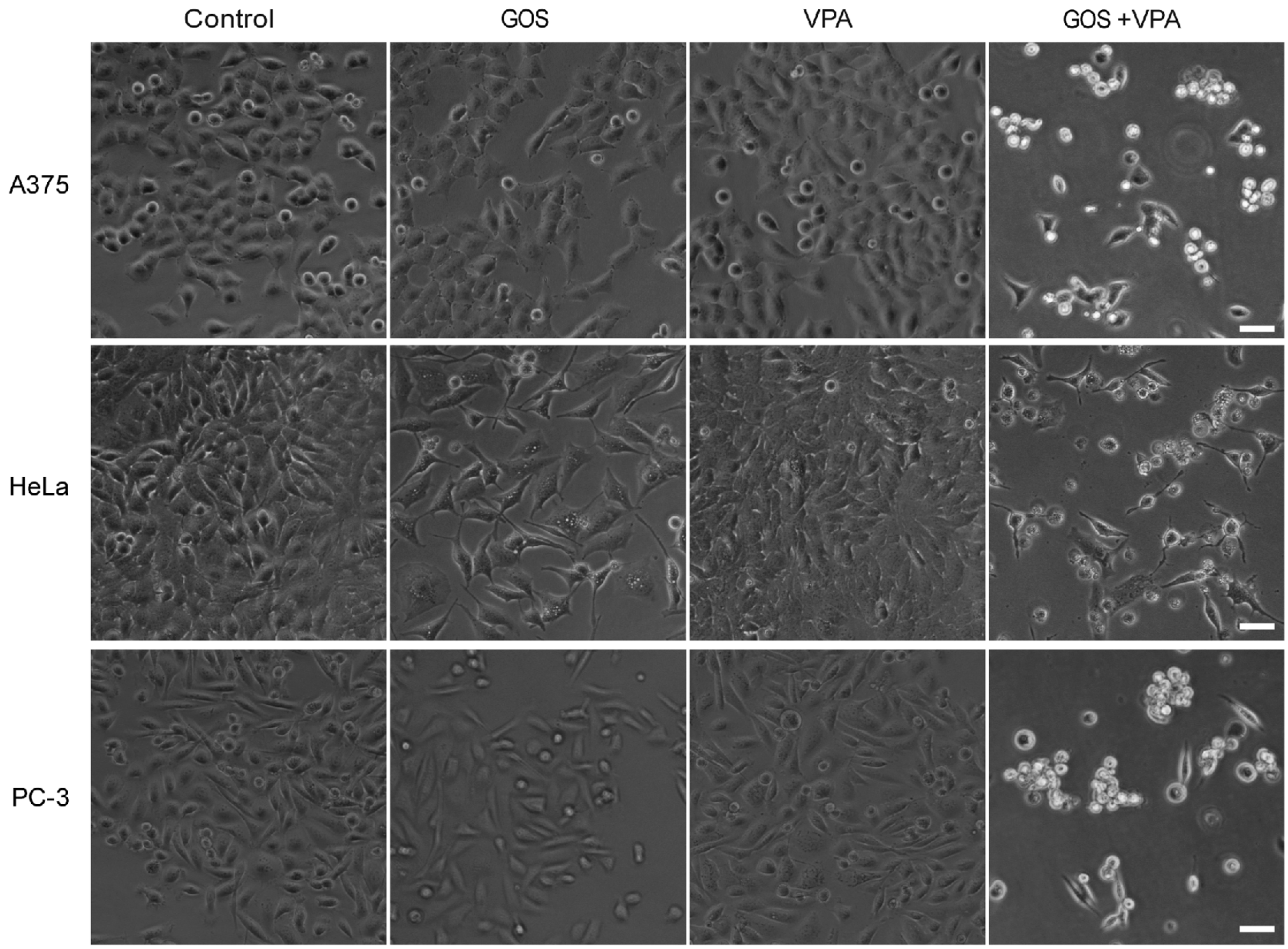

Supplementary Figure S1: The morphology of cells treated with gossypol (GOS), valproic acid (VPA) or their combination. Cells were treated with vehicle (control), GOS (30 μM) and/or VPA (1 mM) for 24 h and their morphology was observed using phase-contrast microscopy (10×). Scale bars, 50 μm.

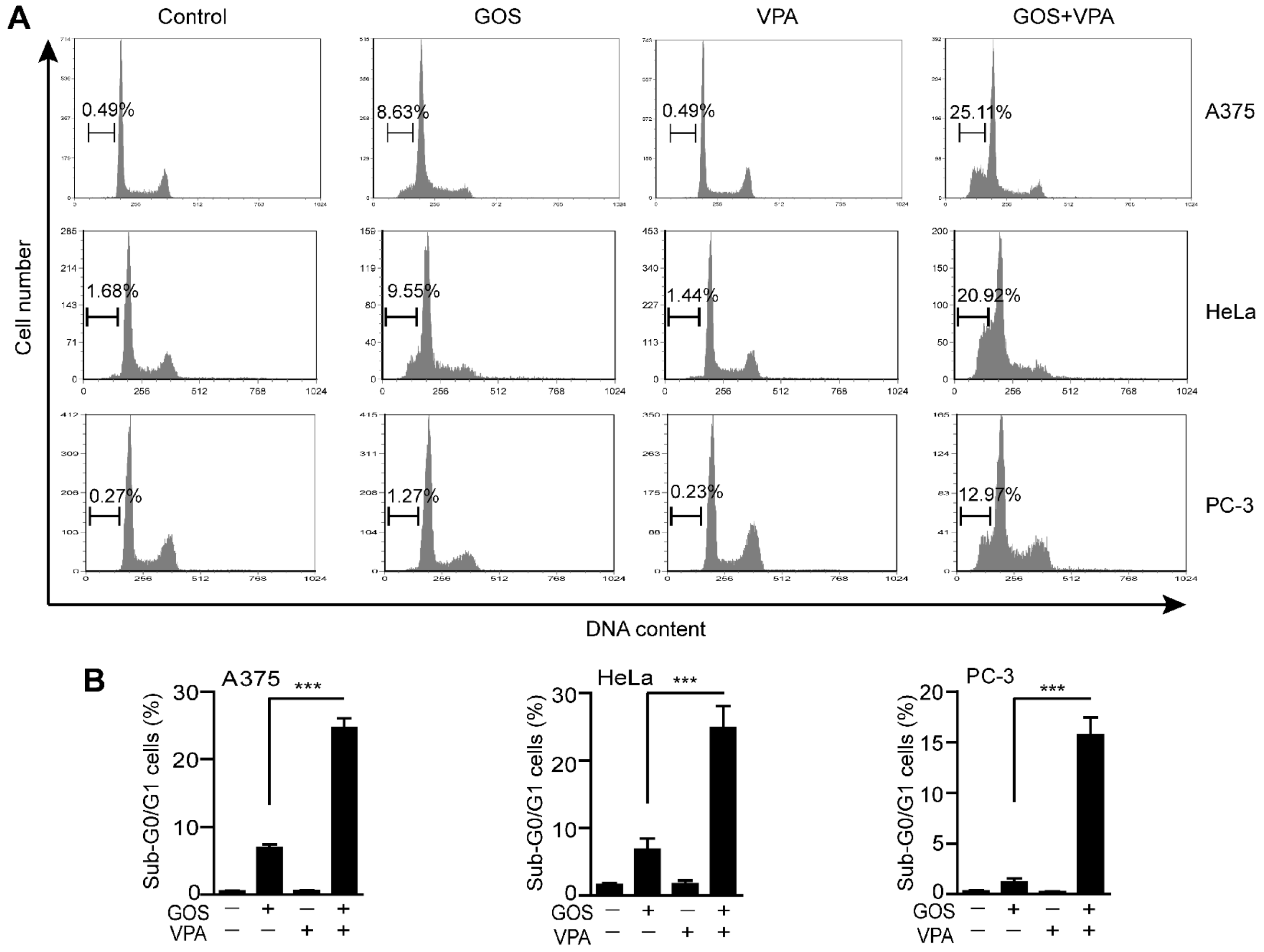

C

Flow cytometry analysis of the effect of GOS and VPA on the cell cycle in A375 cells

Group	G0/G1(%)	S(%)	G2/M(%)
Control	64.61 ± 0.65	13.98 ± 0.88	21.46 ± 0.18
GOS	72.56 ± 0.76**	16.13 ± 1.46	10.42 ± 0.16***
VPA	62.89 ± 1.18	12.81 ± 1.71	23.17 ± 0.66*
GOS+VPA	75.81 ± 0.40***	12.64 ± 0.48	12.48 ± 0.57***

Flow cytometry analysis of the effect of GOS and VPA on the cell cycle in HeLa cells

Group	G0/G1(%)	S(%)	G2/M(%)
Control	64.71 ± 0.89	12.14 ± 0.83	21.88 ± 0.43
GOS	75.41 ± 0.54 **	13.07 ± 1.34	11.51 ± 0.17 ***
VPA	62.91 ± 0.26	12.45 ± 0.46	24.19 ± 1.59 **
GOS+VPA	75.97 ± 0.97 **	12.76 ± 0.37	11.01 ± 0.75 ***

Flow cytometry analysis of the effect of GOS and VPA on the cell cycle in PC-3 cells

Group	G0/G1(%)	S(%)	G2/M(%)
Control	59.48 ± 0.46	11.14 ± 0.39	28.88 ± 0.51
GOS	72.16 ± 0.35 ***	9.98 ± 1.91	17.31 ± 0.32 **
VPA	55.13 ± 0.34 ***	10.01 ± 0.51	34.01 ± 1.19 **
GOS+VPA	64.14 ± 1.87 ***	12.88 ± 0.49	22.68 ± 0.88 *

Supplementary Figure S2: Flow cytometric analysis of cell cycle distribution. A. Cells were treated with vehicle (control), VPA (1 mM), GOS (30 µM), or GOS plus VPA for 24 h, respectively, stained with propidium iodide (PI), and analyzed by flow cytometry. One representative histogram sets of three independent experiments are shown. **B.** Flow cytometric analysis of sub-G_0/G_1 peaks in cells treated with GOS (30 µM) and/or VPA (1 mM). Cells were fixed and stained with PI solution. Percentages of cells in sub-G_0/G_1 are displayed as mean ± S.D. ($n = 3$). ***$P < 0.001$. **C.** Cell cycle distribution of cells treated with vehicle (control), GOS (30 µM) and/or VPA (1 mM) are presented as mean ± S.D. ($n = 3$). *$P < 0.05$, **$P < 0.01$, and ***$P < 0.001$ versus control.

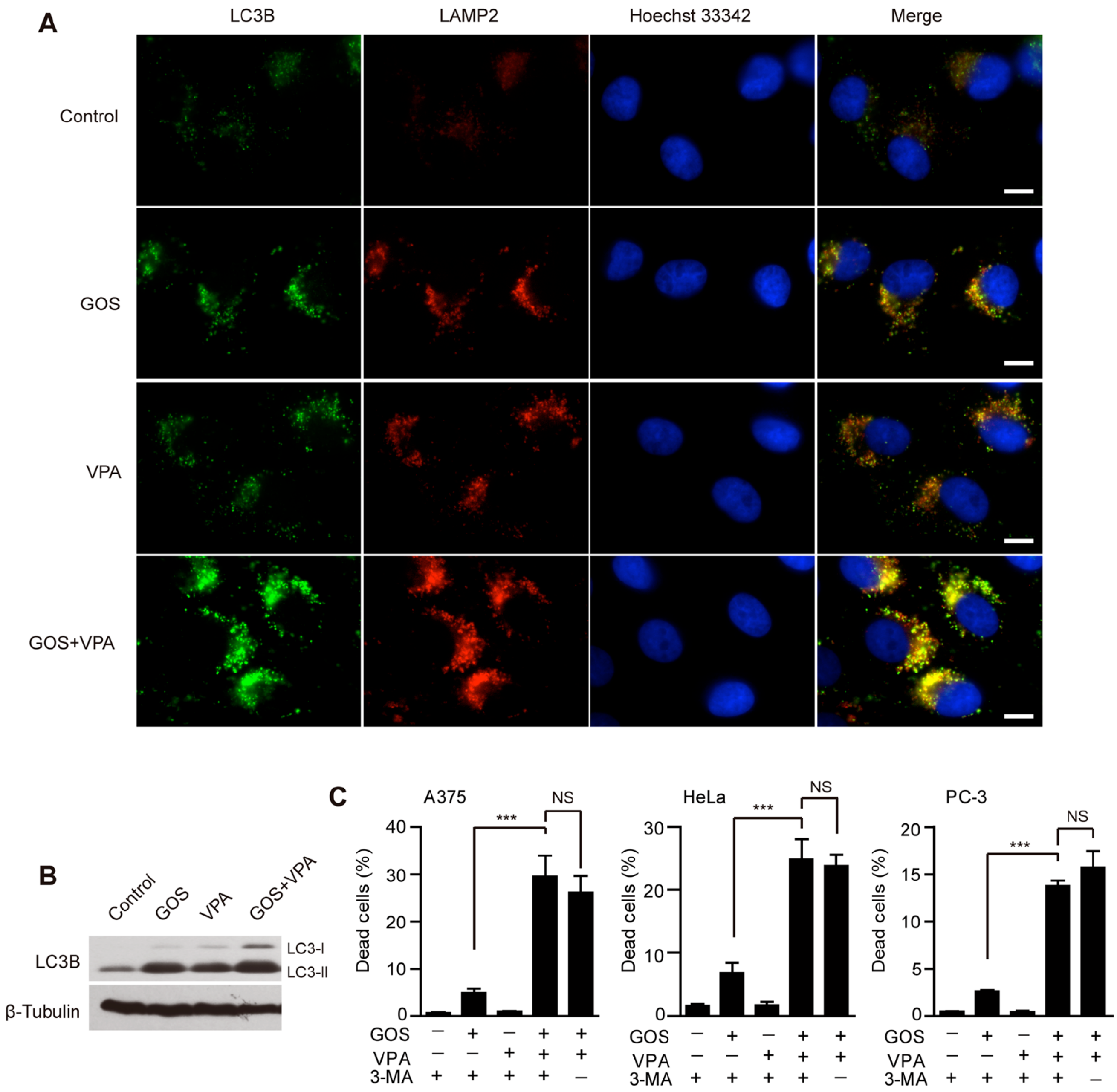

Supplementary Figure S3: The effect of autophagy on cell death induced by GOS and VPA co-treatment. A. A375 cells were treated with control, GOS (30 μM), VPA (1 mM) or their combination for 24 h, respectively. Immunofluorescence was performed using anti-LC3B (green) and anti-LAMP2 (red) antibodies. Co-localization between LC3B and LAMP2 is shown in merged images. Images are representatives of three independent experiments. Scale bars, 10 μm. **B.** Western blot analysis of LC3 levels in A375 cells treated with GOS (30 μM), VPA (1 mM) or their combination for 24 h. **C.** Co-treatment with 3-methyladenine (3-MA, 5 mM), an autophagy inhibitor, failed to suppress GOS+VPA-induced cell death in all tested cells. Values are presented as mean ± S.D. ($n = 3$). NS, non-significant; ***$P < 0.001$.

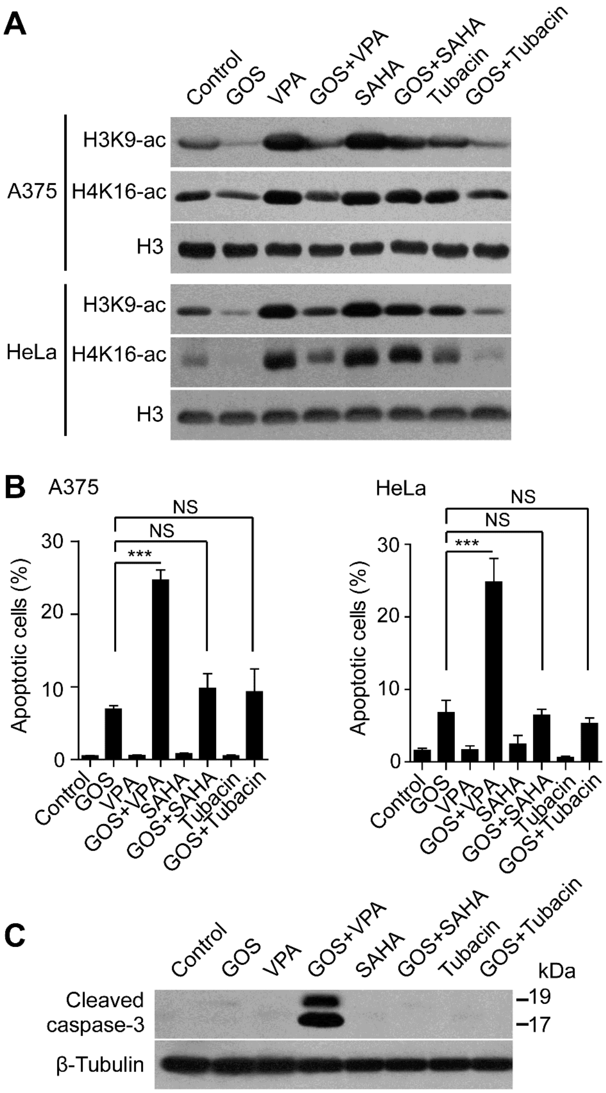

Supplementary Figure S4: Combined effects of GOS and HDAC inhibitors on acetylation levels of histones and apoptosis. A. and **C.** Cell lysates were extracted from cells treated with GOS (30 µM) and/or VPA (1 mM), SAHA (1 µM) and tubacin (2 µM) for 24 h, respectively, and protein expression levels were analyzed by western blotting using specific antibodies. Histone 3 (H3) and β-tubulin were used as a loading control. **B.** Cells were treated with GOS in combination with or without HDAC inhibitors for 24 h and percentages of cells in sub-G_0/G_1 (apoptotic cells), measured by flow cytometry, are displayed as mean ± S.D. ($n = 3$). NS, non-significant; $***P < 0.001$.

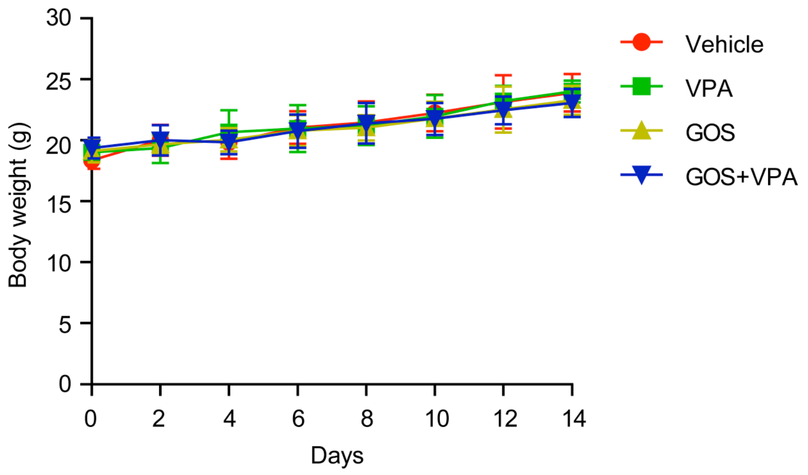

Supplementary Figure S5: Combined effects of GOS and VPA on body weight of nude mice. Mice with human A375 melanoma xenografts were administered intragastrically with vehicle (2% Tween-80 in PBS), VPA (200 mg/kg/d), GOS (20 mg/kg/d) or their combination for 14 consecutive days. Body weight was analyzed (*n* = 6 mice per group).
